# A Gelatinous Pleural Effusion as a Diagnostic Clue

**DOI:** 10.7759/cureus.35942

**Published:** 2023-03-09

**Authors:** Daniela Barroso, Rita Rego

**Affiliations:** 1 Internal Medicine, Centro Hospitalar Universitário de Santo António, Porto, PRT

**Keywords:** thoracocentesis, mesothelioma, gelatinous pleural effusion, pleural effusion, malignant pleural effusion

## Abstract

Pleural effusion is a common presentation of several pathologies, and the determination of its cause is facilitated by macroscopic, biochemical, microbiological, and cellular analysis. A systematic approach to analyzing the fluid allows for a reduction in clinical diagnoses. Only a select number of diagnoses can be established definitively by thoracentesis, including effusions because of malignancy. We report the case of an 84-year-old male with a right large-volume pleural effusion with an initial diagnostic thoracocentesis demonstrating an exudate with a gelatinous appearance and exudate characteristics. The physical characteristics of the pleural effusion quickly raised the suspicion of mesothelioma, a rare tumor associated with a poor prognosis. In most diseases related to pleural effusion, fluid analysis yields important diagnostic information, and in certain cases, fluid analysis alone is enough for diagnosis. Malignant pleural mesothelioma may present as a viscous pleural effusion with gelatinous characteristics, which may immediately raise suspicion and contribute as a diagnostic clue in the initial study of a pleural effusion. This article was previously presented as a meeting abstract at the 28º Congresso Nacional de Medicina Interna in October 2022.

## Introduction

Excess fluid accumulation in the pleural space can be caused by both malignant and benign conditions [1]. Usually, pleural effusions are classified into transudates and exudates [1]. An exudate occurs when there is an increased vascular permeability [2], and more investigations are usually required to identify the cause and guide specific therapy [1]. The patient's medical history will help determine the working differential diagnosis and should include data such as the patient's medications, risk factors, and symptoms that could point to respiratory infections, malignant diseases, autoimmune diseases, heart, liver, and renal conditions, as well as other conditions [1]. Having a complete occupational history and asking detailed questions concerning asbestos exposure is crucial: even a small amount of asbestos exposure can result in mesothelioma, which develops decades later [3]. When making a clinical choice to determine an appropriate treatment for pleural effusion management, the composition of pleural fluid may be crucial. The fluid analysis provides significant diagnostic information for most pleural effusion-related disorders, and in some circumstances, the diagnosis can be made only based on the fluid analysis. Several complementary examinations that can help with the identification of common and uncommon pleural effusions are explained, along with the numerous noteworthy features of pleural fluid [4]. When first considering this pathology, further studies and diagnostic procedures can be carried out for an early diagnosis.

## Case presentation

**Figure 1:**
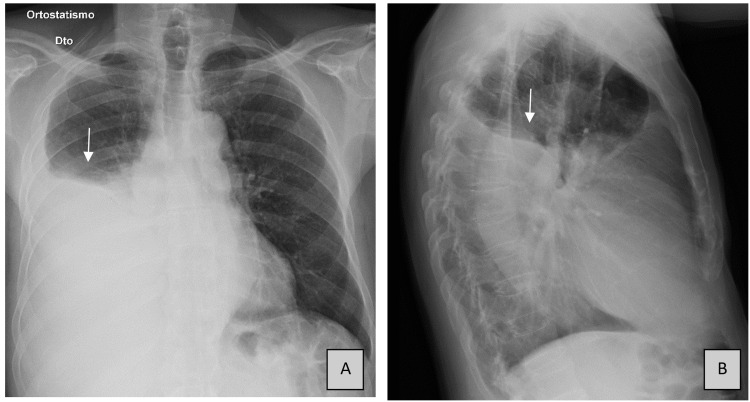
Chest radiographs. Posteroanterior (panel A) and lateral (panel B) chest radiographs presenting a large right pleural effusion (arrows). Dto: Right.

An 84-year-old man with a 25-year history of smoking and chronic bronchitis presented to an ED with complaints of fatigue, malaise, dyspnea, and pleuritic chest pain for one month. He lived in a rural area of Porto in Portugal and was a retired construction materials truck driver. He denied fever, cough, having contact with ill people, edema of the lower limbs, or orthopnea. On arrival, blood pressure was 138/78 mmHg, heart rate was 76 beats per minute, the temperature was 36°C, and the patient was saturating at 93% on room air. The physical exam was remarkable for absent breath sounds in the lower two-thirds of the right lung field, and the BMI was 19 Kg/m^2^. The white-cell count was 7800/mm3, the hemoglobin was 15.6 g/dL, and the platelet count was 182,000/mm^3^. His basic metabolic panel and the C-reactive protein were normal (2.66 mg/L). He presented with type 1 respiratory failure (pO_2_ of 59 mmHg), and a right large-volume pleural effusion was visible on his chest radiography (Figure 1). He was hospitalized to be further investigated and for diagnosis clarification.

An initial diagnostic thoracocentesis was performed, and 1500 mL of yellow, gelatinous fluid was removed (Video 1). Regarding the analysis of the pleural fluid, the fluid glucose level was 15 mg/dL (serum levels 84 mg/dL), the protein level was 4.4 g/dL (serum levels 7.2 g/dL), the lactate dehydrogenase level was 824 U/L (serum levels 195 U/L), and the pH was 7.1. Adenosine deaminase was normal (18U/L). The cytologic analysis revealed a predominance of monocytes (1506 cells/uL), lymphocytes (476 cells/uL), and macrophages (1748 cells/uL). There were still other cells of a more immature and large appearance. Gram staining of the pleural fluid revealed no organisms, and acid-fast bacillus tests (smear, polymerase chain reaction, and culture) were negative.

**Video 1:** Macroscopic characteristics of the pleural effusion. A yellow, gelatinous fluid was removed from the pleural space of the patient.

**Figure 2:**
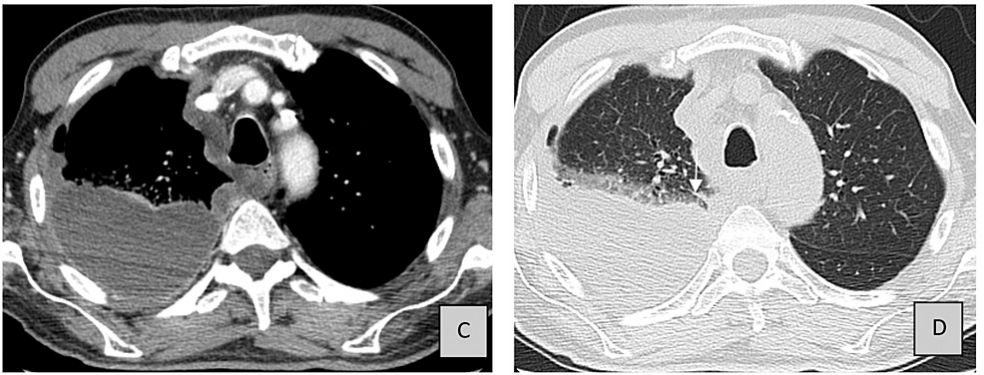
CT of the thorax. A contrast-enhanced axial CT image of the thorax, transverse cut (panels C and D), presenting a large pleural effusion and thickening of soft tissues, involving the anterior and superior right hemithorax, insinuating into the mediastinum, with involvement of the pleura mediastinal on its right side.

Pleural tuberculosis was excluded, and the investigation continued with a thoracoscopy with the identification of a voluminous pleural mass extending to the parietal and diaphragmatic pleura, very suggestive of a malignant process. The CT of the chest supported the diagnosis (Figure 2).

A pleural fluid sample was sent for pathological anatomy analysis, and biopsies of the mass observed in the pleura were performed. The anatomopathological study of the thoracoscopic biopsy products showed fragments of fibrous tissue infiltrated by a population of epithelioid cells with marked cytological atypia organized in nests and isolated cells. The aspects described support the diagnosis of epithelioid mesothelioma. The oncology team was then involved in the case, and palliative systemic treatment was started. The patient passed away two years after the diagnosis in the context of the progression of the disease.

## Discussion

Pleural fluid viscosity is influenced by its cellular constituents and macromolecule composition [5], and gelatinous pleural effusion can represent a diagnostic and therapeutic challenge. Mesothelioma, melanoma, lung cancer, myxoid sarcoma of the pleura, multiple myeloma, and abdominal or gynecologic cancers (including pseudomyxoma peritonei) have been linked to a malignancy-related gelatinous pleural effusion [6]. The slimy appearance of pleural mesothelioma is thought to result from the hyaluronic acid that it produces [6]. The extracellular matrix contains a large amount of hyaluronic acid, a common high molecular weight linear glycosaminoglycan [7] with diagnostic and prognostic importance for mesothelioma [8]. Hyaluronic acid has been found in the pleural fluid of various malignant and nonmalignant diseases [9]. However, in contrast to other diseases, malignant mesothelioma produces more hyaluronic acid, which is thought to contribute to the viscous appearance of the pleural effusion [10]. Therefore, elevated levels of this substance in the pleural fluid that is translated by the macroscopic aspects support the diagnosis of mesothelioma [10]. Pleural mesothelioma is a rare and aggressive cancer of the pleural surface, associated with poor prognosis [11]. Malignant pleural mesothelioma's subtle and frequently cryptic clinical signs and symptoms cause the diagnosis to be delayed despite repeated assessments of the patient pleural fluid [12]; hence diagnostic clues such as the physical characteristics of the pleural fluid are essential for its early identification. The most frequent initial symptom of pleural mesothelioma is malignant pleural effusion [13], which may be asymptomatic or have few symptoms and totally disappear. However, the pleural effusion may be recurring, hemorrhagic, and filled with a significant amount of fluid [14,15]. Cytological investigations for malignant pleural mesothelioma may reveal the presence of atypical cells [16]. Malignant pleural effusion currently has no known treatment; several therapeutic approaches have been tried with variable success rates [17]. As with other types of malignant pleural illnesses, pleural mesothelioma therapy focuses mainly on symptom palliation [18].

## Conclusions

Pleural mesothelioma is rare cancer, and its diagnosis is essential for an early and timely orientation of the patient. In most diseases related to pleural effusion, fluid analysis yields important diagnostic information, and in certain cases, fluid analysis alone is enough for diagnosis. Malignant pleural mesothelioma may present as a viscous pleural effusion with gelatinous characteristics, which should immediately raise suspicion and contribute as a diagnostic clue in the initial study of a pleural effusion.
